# Exploring Knowledge and Perspectives of South Asian Children and Their Parents Regarding Healthy Cardiovascular Behaviors: A Qualitative Analysis

**DOI:** 10.1177/2333794X20924505

**Published:** 2020-07-01

**Authors:** Adeleke Fowokan, Kaitey Vincent, Zubin Punthakee, Charlotte Waddell, Miriam Rosin, Navjot Sran, Scott A. Lear

**Affiliations:** 1 Simon Fraser University, Burnaby, British Columbia, Canada; 2 McMaster University and Population Health Research Institute, Hamilton, Ontario, Canada; 3 University of British Columbia, Vancouver, British Columbia, Canada; 4 Providence Health Care, Vancouver, British Columbia, Canada

**Keywords:** South Asians, cardiovascular disease, physical activity, diet

## Abstract

South Asian children and parents have been shown to have a higher risk for cardiovascular disease (CVD) relative to white individuals. To design interventions aimed at addressing the comparatively higher burden in South Asians, a better understanding of attitudes and perspectives regarding CVD-associated behaviors is needed. As a result, we sought to understand knowledge about CVD risk in both children and parents, and attitudes toward physical activity and diet in both the children and parents, including potential cultural influences. In-depth interviews were conducted with 13 South Asian child-and-parent dyads representing a range of child body mass index (BMI) levels, ages, and with both sexes. South Asian children and parents demonstrated good knowledge about CVD prevention; however, knowledge did not always translate into behavior. The influence of social and cultural dynamics on behavior was also highlighted. To ensure that interventions aimed at this population are effective, an understanding of the unique social dynamics that influence diet and physical activity–related behaviors is needed.

## Introduction

South Asian ethnicity refers to individuals who have ethnic roots in the countries that collectively make up the Indian subcontinent. These countries include Bangladesh, India, Nepal, Sri Lanka, and Pakistan. South Asian adults have been shown to have a significantly higher prevalence of cardiovascular disease (CVD), diabetes, hypertension, and body fat when compared with their white counterparts.^1^ Of interest, this observation also holds true for young South Asian children as studies have also documented a higher prevalence of hypertension and higher body fat relative to white children.^2,3^ South Asian children and adults also tend to have lower levels of physical activity, higher consumption of dietary fat, and a limited knowledge of CVD risk and health practices,^4,5^ suggesting that significant potential for CVD risk reduction in South Asians might lie in targeting behavior.

Lifestyle intervention studies aimed at addressing the burden of CVD in South Asians in primary care settings have demonstrated modest promise in weight, blood pressure, and cholesterol reduction.^6^ However, it remains unclear how effective these interventions are in altering behavior in the long term. Additionally, studies conducted in both South Asian children and adults have shown that South Asians report significant barriers to behavior change.^7^ For example, one qualitative study in the United Kingdom found South Asians to report cultural barriers to physical activity uptake and diet change.^8^ Comments such as “I can’t leave our food, this is what I have been eating since I was born and is what I will eat until I die” and “I would like to swim, but as yet have not found a place where I will be allowed to swim with my Kirpan [religious dagger]” were used to describe barriers to diet change and physical activity uptake,^8^ respectively. In addition, one review of Canadian studies found that barriers to physical activity in adults were related to cultural beliefs that attribute illnesses to factors outside of individual control and a perceived lack of time due to family and work commitment. In children, barriers to physical activity were attributed to culture that emphasizes academic achievement in children, resulting in children prioritizing study time over physical activity.^9^

A key aspect in creating effective health education and behavior change interventions is the understanding of cultural or individual beliefs and perspectives around health-related behaviors to enable the tailoring of more proactive and effective interventions.^10^ The few studies that have sought to understand cultural barriers to behavior change in the South Asian population have focused primarily on the individualized perspective of children and adults alone without exploring the combined perspectives of children and their parents. Given that a disproportionate burden of CVD-related outcomes have also been found in South Asian children^2,11,12^ and that CVD-related behaviors in children such as diet and physical activity might be influenced by parental behaviors and cultural factors, understanding this combined perspective seems vital.

### Theoretical Framework

Increasingly, research studies focused on promoting health and health interventions in ethnic minorities are becoming more aware on the need for cultural sensitivity in the implementation of these programs. Yet, important questions remain about the right approaches to implementing cultural sensitivity when seeking to improve the health behaviors of minority groups. While different approaches have been proposed, the presence of a consensus exist regarding the need for addressing deep-rooted social and cultural factors that influence the health of ethnic minorities. This understanding appears to be particularly exemplified in the study by Resnicow et al,^13^ which we have adopted as a framework to guide data collection, analysis, and interpretation.

The framework by Resnicow et al^13^ was originally proposed to help guide the development of interventions by distinguishing interventions aimed at promoting healthy behavior in ethnic minorities at varying degrees of cultural sensitivity. However, as this study sought to understand the range of influences on the behavior of South Asians, we believed this could also be adapted for use in understanding the range of influences on South Asian’s health behaviors. Hence, we chose to utilize this framework to ensure that our qualitative process was culturally rooted. Resnicow et al define cultural sensitivity using 2 dimensions: surface and deep structures. Surface structures delineate the need for connecting health promotion messages with identifiable characteristics such as dietary preferences or physical activity preferences of the target audience with the aim of improving uptake.^13^ Deep structures, in contrast, refer to the integration of sociocultural, environmental, and psychological forces that influence the targeted behavior of the specific population being studied.^13^ Resnicow et al^13^ outlines strategies and actions to take to ensure that the qualitative process is culturally rooted, including conducting a pretest of the questions on a sample group to ensure cultural relevancy and “exploring the thoughts, feelings, experiences, and so on of participants regarding the target behavior as well as environmental enabling or constraining factors.” This helps ensure the receptivity of the message and also helps ensure that the findings obtained are of cultural importance to the health of the participant group. Furthermore, understanding what role culture and related factors play in influencing the health behaviors of South Asians will also be essential in informing the design of effective health promotion and other intervention programs.

### Study Objective

The present study focuses on understanding (1) knowledge about CVD risk in both children and parents and (2) attitudes toward physical activity and diet in both the parents and children, including potential cultural influences.

## Methods

### Study Design

We recruited child (age range = 10-18 years) and parent dyads by contacting individuals who had previously participated in the Research in International Cardiovascular Health–Lifestyles, Environments, and Genetic Attributes in Children and Youth (RICH-LEGACY) study. The RICH-LEGACY study was a cross-sectional investigation of risk factors for CVD among South Asian children in Canada. During recruitment, participants were asked if they will like to be contacted for a future follow-up qualitative study. Study participants were recruited from the pool of RICH-LEGACY participants who had agreed to be contacted for a follow-up qualitative study.

In addition to recruiting individuals from the RICH-LEGACY study, a small number of participants (both children and their parents; n = 4) who did not take part in the RICH-LEGACY study were also recruited. These children were recruited by a South Asian research assistant who had established rapport with the South Asian community members. This was done to ensure that diverse viewpoints were obtained, including from participants who did not contribute to the RICH-LEGACY study findings. We utilized purposive sampling to recruit children from a range of age groups, body mass index levels, and with both sexes to ensure representation across the demographic spectrum. To ensure that non–English-speaking parents were included, interviews were available in Punjabi. We chose to recruit children older than 10 years because that threshold provided us with children who were knowledgeable, cognitively aware, and competent in communication to avoid issues like the reliance on parents for responses to questions, which have been reported in children of younger ages.^14^

### Data Collection

In-depth interviews were conducted with South Asian children and their parents over a 4-month period in 2018 using a semistructured interview guide that was developed based on the theoretical framework described in the introduction and similar qualitative studies in the South Asian children and adult population (Supplementary file, available online). To avoid parental influences on child responses, we interviewed children and parents separately. The interview guide explored questions related to physical activity and diet, and relevant sociocultural factors including the influences of the school, social, and cultural environment, which might influence these behaviors. The interviews were conducted until data saturation (ie, the point where no new codes were originating from the interviews) was attained. Data analyses was ongoing and iterative throughout the interview process, which helped inform the point at which data saturation was achieved. Participants were interviewed over the phone (n = 9 child-and-parent dyads) or in-person (n = 4 child-and-parent dyads) based on participant preference. Two interviews were conducted in Punjabi and the remainder in English. Each separate interview conducted lasted on average between 25 and 40 minutes. The first author, who has a background in health sciences and an awareness of potential issues with conducting research in children, conducted 9 interviews. The research assistant, with valuable research experience, who was first trained on potential issues with conducting research in children, conducted 3 interviews, including the interviews in Punjabi. One interview was conducted jointly. Translation from Punjabi to English was conducted by the research assistant.

### Data Analysis

Interviews were digitally recorded and transcribed verbatim. For interviews conducted in Punjabi, the interviews were translated and transcribed into English by a research assistant fluent in both languages. Following this, the interview transcripts were reviewed several times in order to gain familiarity on the subject matter. We utilized both inductive and deductive coding to perform a thematic analysis of the data.^15^ This was done by firstly generating roughly developed codebooks to guide the coding process using existing literature evidence. This codebooks were then refined as the coding process went on, and in cases where portions of the interview transcripts were not captured by existing codes, new codes were developed from scratch. The coding process was iterative, seeking to add and revise codes that emerged from all the interviews in order to capture the totality of the participants’ views. After multiple coding iterations were completed, axial coding was utilized to identify relationships between codes, allowing for the development of common themes reflected throughout the data. To ensure that data analysis was driven by the theoretical framework, all of the developed themes were reviewed to ensure overall coherence of themes with the theoretical framework. In cases where there were discrepancies, themes were reviewed and merged with other suitable themes. The coding process was done separately for the child and parent interviews. However, child codes were constantly compared with parent codes to highlight similarities and differences in themes for both groups. The transcripts were reread multiple times until no new themes were identified. Coding was also completed by 2 independent researchers (AF and KV), and interrater reliability was calculated using the weighted Cohen’s κ and found to be 0.77. Disagreements in the coding process were resolved by discussion until consensus was reached. To allow for broad representativeness in participant views, outliers—or cases which contradicted the ideas emerging from the data—were identified and incorporated in the analysis. Data analysis was aided by NVivo 14 (QSR international, Melbourne, Australia).

### Ethical Approval and Informed Consent

This study was approved by the Simon Fraser University (Approval # 2011s0311) and Providence Health Care (Approval # H15-00960) Research Ethics Boards (REBs). Parents provided written informed consent prior to enrollment in the study and signed consent forms on behalf of their children, while children assented to take part in the study.

## Results

Four key themes emerged from the data analysis: knowledge about the role of behavioral factors in CVD prevention, attitudes toward diet, attitudes toward physical activity, and the impact of influential actors/medium on health behaviour. We also highlighted subthemes for each theme to allow for easy comparison between children and parents. Below, we summarize these themes in more detail, together with illustrative quotes from participants, edited to ensure clarity. Table 1 provides an overview of the participant sociodemographic characteristics.

**Table 1.** Sociodemographic and Anthropometric Distribution of Child-Parent Dyad.

Characteristic	Child-Parent Dyads (N = 13)
Child age	14.1 ± 2.2
Child sex, n (%)	
Female	5 (38%)
Male	8 (62%)
Children’s BMI categories, n (%)	
Normal weight	6 (50%)
Overweight	3 (25%)
Obese	3 (25%)
Parent’s sex, n (%)	
Female	8 (62%)
Male	5 (38%)
Parent’s highest education, n (%)	
No formal education	0
Primary secondary school	4 (31%)
Trade school	4 (31%)
College/University	5 (39%)

Abbreviation: BMI, body mass index.

### Theme 1: Knowledge About the Role of Behavioral Factors in Cardiovascular Disease Prevention

#### Children

Most child participants were aware of the beneficial effects of a healthy lifestyle, highlighting the combined role of diet and physical activity in addressing the higher risk of CVD in South Asians. One of the comments was, “I think exercising and eating healthy are the best ways to reducing heart disease in both children and adults” (RL6: Male, age 14). Some children also suggested the role of broader well-being in addressing CVD risk. For example, one child highlighted the impact of stress on cardiovascular health: “Don’t be stressed a lot. Being positive also helps in keeping your heart healthy” (RL11: Female, age 13).

#### Parents

Similar to the children, most parents interviewed in this study were aware of the higher CVD risk in the South Asian population, noting the role of lifestyle factors for the increased risk. For example, one parent highlighted the impact of diet and sedentariness on the cardiovascular health of South Asians: “I think the lack of exercise and not eating healthy foods like vegetables and fruits is the number one reason for the high risk in the South Asian population” (RL13). When asked what needed to be done to address this risk, most parents highlighted the importance of physical activity and healthy diets. However, one parent also surmised the influence of changes in geographical location to CVD risk-related behavior in South Asians:

“South Asian foods are not very healthy like rich in vegetables, rather we eat foods that might be high in sugar-like rice, sugary foods, and foods processed from wheat. In Asia where we come from, we eat a lot of these things but we do physical activity to offset their impact. Here, people sit for long hours and there is less activity, so they have to eat less of the unhealthy things and prioritize healthy food. (RL9)”

As well, some parents highlighted the importance of broader holistic approaches to addressing CVD risk in South Asians. One of the comments was, “I believe praying, and yoga also help and are important for cardiovascular health” (RL3).

### Theme 2: Attitude Toward Diet

Given the specific role that diet plays in the development and progression of CVD risk factors and the strong cultural component attached to diet, we questioned participants about their attitudes toward diet. This included their knowledge about healthy foods, the South Asian diet, and barriers toward healthy eating.

#### Children

##### Knowledge about healthy diets in children

When asked to define what a healthy diet was, the children provided conceptual and technical definitions of a healthy diet. These included details such as the food guide and the major food groups specified in the food guide and their servings, for example, “The right servings of all the food types—fruit and veggies, dairy product, and meat product—that is contained in the food guide” (RL2: Female, age 12). One child also raised important considerations for tailoring dietary servings to individual metabolic needs:

“The healthy diet is what we learn about Canada’s Food Guide. You need 5-12 servings daily and only 2-3 of meat and alternatives, 5-10 of fruits and vegetables, but these serving sizes depend on the person and how active they are. (RL10: Male, age 13)”

Despite the nutritional awareness that children showed, there was a mixed response when they were asked to self-assess the quality of the diet that they consumed. For example, one child stated:

“I think generally speaking the food I eat at home is healthy. My parents make sure that we eat good healthy foods at home. When I am out with friends though, I sometimes feel pressured to eat junk foods. (RL6: Male, age 14)”

##### Barriers to healthy eating in children

For children who self-assessed their food as unhealthy, we questioned them on why given their understanding of the importance of healthy foods they still ate unhealthy foods. Most children documented the appealing taste and flavor of the unhealthy foods as the biggest barrier to healthy eating. Comments illustrative of these findings included the following:

“The thing that stops me from eating healthy is the fact that the foods I eat taste better and have more flavor and texture than the healthy foods that are given to me. (RL8: Female, age 16)”

and

“You know with my type 1 diabetes sometimes I will look at people eating certain things and say to myself why can’t I do that also. So maybe once in a while, I will treat myself out but I can’t really do that all the time. (RL4: Male, age 15)”

##### Motivation of healthy eating in children

For children who self-assessed their diet as healthy, we questioned them on motivations for dietary choices. A range of motivations for healthy eating were highlighted. Specifically, one child raised concerns about not wanting to be bullied for their weight:

“I think at school, there are a lot of people who do not look healthy and, you know at school sometimes you get bullied if you’re fat and I do not want to be that person to get bullied, so I keep eating healthy. I do not want to gain a lot of weight. (RL13: Female, age 14)”

In addition, one child with diabetes also mentioned the presence of the health condition as a motivator for maintaining healthy dietary practices.

#### Parents

##### Knowledge about healthy diets in parents

Most parents demonstrated good general nutritional awareness when asked to explain what healthy eating meant, referring to Canada’s food guide in some cases as the standard to follow. For example, one parent said, “Enough fruits and vegetables, fair amount of proteins in it, limiting sugary things, you know, Canada food guide kind of recommendation” (RL2). Some parents also mentioned the need for dietary diversity (ie, including a variety of foods): “Vegetables, fruits, meats including seafood, and dairy product. I think for the meats, reducing red meat consumption and probably replacing them with chicken or seafood is recommended. And yes, our household diet meets those requirements” (RL1), while another stressed the importance of checking food labels before purchasing foods.

##### Perception of the South Asian diet in parents

There were mixed responses from parents about the healthiness of the South Asian diet. Some parents highlighted the potential issues with the traditional diet, especially those related to its high saturated fat content. A few parents, however, highlighted important variations in the South Asian diet, for example, “The traditional South Asian diet is healthy because you are not eating a lot of fried food. We use less oil than Indian and Pakistani food and it’s not too much spicy, and use a lot of vegetables” (RL11). One parent also alluded to specific dietary restrictions practiced by the family and how that might improve the dietary quality of the South Asian diet:

“I think that varies from family to family. Our version of the South Asian diet is healthy. We are vegetarian so we only eat lots of vegetables, low-fat milk, whole wheat foods, and we try to use less oil and when we do we use low fat butter and reduce lots of frying. (RL7)”

##### Barriers to health eating in parents

One parent acknowledged the challenges with behavior change as it relates to food for South Asians: “I will say South Asians really like sweets and they also do not want to give up their butter. . . . I can’t give up my butter either” (RL1). A few parents alluded to the impact of what was termed “fast lifestyle” on the dietary choices of families: “Just a fast life-style, less time to cook the food prevents my family from eating healthy. Sometimes the children are also picky and don’t like home-made Indian food” (RL10). In addition, one participant highlighted the role of family structures and the challenges it poses to influencing health behavior:

“So, if it is a typical South Asian or Indian family with older generations living in the household then I think the food might need some work because there will probably be more fats, oils, and fried foods. But if there is no grandparents around and the family has been here for a while, and they are aware, then I think it is good. . . . Because it is very hard to change the habits of older people. (RL2)”

It appears that family dynamics might exert influences on dietary-related behavior, as older family members might not be as amenable to change as younger family members, as such, their presence in households might make it harder to make healthier changes to meals.

##### Motivation for healthy eating in parents

Health reasons appeared to be the strongest motivator for healthy eating. For example, one parent stated:

“. . . I will say after the doctor diagnosed me with high cholesterol. That was a few years ago and I realized I do not want to suffer that again because it could lead to a heart attack and then I started changing after that. We have also been pretty much eating healthy after our kids. When we first got married, we both were working 7 days a week and didn’t care about food but after we had our kids we decided to be more careful to make sure they eat healthy food. (RL13)”

In particular, families with a history of CVD risk appeared to be more amenable to dietary changes. For example, “My biggest motivator for being health conscious especially with what I eat and how active I am is because my parents had issues with their health—high blood pressure problems and other complications” (RL6).

### Theme 3: Attitude Toward Physical Activity

#### Children

##### Physical and mental health benefits of physical activity in children

Children demonstrated a good level of knowledge about the importance of physical activity, highlighting its physical and mental health benefits. Its impact on improving focus in school was also mentioned. Comments illustrative of these findings include:

“Yes, physical activity is important. Besides the fact that it can help prevent diseases and keep people fit, for me, I feel better mentally every time I take part in physical activity like playing basketball with my friends . . . it keeps my mind awake. (RL6: Male, age 14)”

and

“I find that it helps me a lot to focus during the school as well as calming me down. (RL13: Female, age 14)”

##### Motivators for physical activity in children

The school environment was frequently highlighted as one of the biggest influences on children’s physical activity behavior: “I am active at school because we have PE almost every day, and sometimes in the middle of class, the teachers ask us to get up and stretch (RL11: Female, age 13). A few students also commented on the positive influence of peers on physical activity uptake. For example, one child stated:

“I like to play sports, mostly basketball, and it is more fun to play with friends than play alone. I think friends motivate you to take part in physically active because when I am by myself at home, I mostly watch TV. (RL10: Male, age 13)”

##### Barriers to physical activity in children

Children cited a range of barriers that affected their levels of physical activity. These barriers involved the excessive use of technological mediums such as TV, video games, and mobile phones. For example, one child stated: “Sometimes when I am playing video games, I do not want to go outside to play with my friends” (RL9: Male, age 11). Another child mentioned: “My phone definitely stops me from being physically active because once I start to do something on my phone, I don’t realize how long I have been doing that one thing” (RL8: Female, age 16).

#### Parents

##### Religious and cultural influences on physical activity in parents

Given that previous studies have reported religious and cultural barriers to physical activity uptake, some of which include the inability to wear parts of the religious attire in conventional recreational spaces such as swimming pools, we questioned parents about the influence of culture and religion on their choice of physical activity. The comments provided by parents suggested that the influence of religion and culture on physical activity appear to be less salient in this group of South Asians:

“For me, I do not believe religion and culture play a role in the type of activity I engage in. Would I participate in something like Bhangra (a traditional South Asian dance) if I had the opportunity? Yes, but that’s because dancing is my hobby and I like to get involved in something that I like to do and if it gets me active then count me in. (RL6)”

One parent, however, suggested that cultural differences in the country in which one was raised might influence physical activity uptake:

“I think of when we were growing up (in Asia), there was lots of physical work around, lots of walking was included, so, the exercise was never really taught as something that you should add into your day. So, we grew up seeing that, where no one really talked about exercise because we didn’t really need exercise around. The housework and everything we did was already burning extra calories and all. So, it is kind of sometimes challenging to make changes to that mindset. (RL8)”

##### Motivators for physical activity in parents

Health reasons were given as motivations for participation in physical activity. For example, one parent mentioned,

“Your health proves important to an individual participating in physical activity. For instance, I have high blood pressure problems; however, I have noticed that when I go walking, it helps with my blood pressure so this is an activity that I do more often. (RL3)”

One parent also highlighted the communal camaraderie gotten from participating in physical activity with groups of other women and how that might positively influence the uptake of physical activity: “. . . groups with other women, help me be physically active. Even other people when I walk around the neighborhood, it allows you to do more than just exercising and you can catch up with friends” (RL10).

##### Barriers to physical activity in parents

Parents also documented a range of barriers to the uptake of physical activity. The most commonly highlighted of these barriers were lack of time and the presence of certain health conditions, which might act as impediment to the uptake of physical activity. Comments illustrative of this includes: “Time is the main factor. Just busy with work and the kids stops me being physically active” (RL11) and “The biggest barrier for me is because I have shoulder pain. Another thing is my feet swell up, and get tired, and eventually hurt” (RL5).

### Theme 4: Impact of Influential Actors/Medium on Health Behavior

In addition to the other themes already discussed, the impact of influential actors/medium were underscored throughout the interviews by both children and their parents. These factors relate to a range of worthwhile influences on behavior that could positively or negatively affect CVD-related behaviors. For children, these factors included social media influences on behavior, while for parents the factors discussed highlight the positive impact of health practitioners on behavior.

#### Children

##### Influence of social media on behavior in children

The connections between social media, societal influences, and advertising appeared frequently as important factors in terms of negative effects on behavior change. Specifically, some children raised noteworthy concerns regarding the influx of advertisements on social media platforms that portray unhealthy habits as fashionable: “I think sometimes social media has a negative effect on behavior in children. For example, smoking is presented on social media as a good thing and that can affect children’s habits, same for junk food” (RL2: Female, age 12).

However, some children also highlighted the potential of social media in promoting healthy behavior. For example, one child mentioned:

“Sometimes it can help motivate you. Like, I like LeBron James and if I saw him eat healthy, I might do the same too because I think he is successful at what he does and I want to be successful like him. (RL12: Male, age 14)”

#### Parents

##### Influence of doctors/health professionals on behavior in parents

Some participants highlighted the positive role played by doctors in improving personal health behaviors:

“. . . the family doctors should mention things like exercise and eating healthy to people a little bit more maybe and give them a hard time. I always used to be scared when I go to my family doctor because she is so strict (laughs). She tells me that I have to watch out and I always listen to her. (RL13)”

One participant also suggested targeting South Asians for health promotion at religious centers:

“We can have the workshops around, people (health specialists) should come to the Gurdwara (our temples), they can give brochures to people there and educate them about diet and how to cook healthy foods. I think that is a really good start, especially for the elderly, having it in their language also, discussing what food is good for you and what is not good for you. (RL12)”

## Discussion

We sought to explore knowledge about CVD risk in both children and parents, and attitudes toward physical activity and diet in both the children and parents, including potential cultural influences in a population of South Asian children and their parents. Four relevant themes were identified for both children and parents. Broadly, these 4 themes document the range of influences on health behaviors for both children and their parents, specifying potential barriers, motivators, and relevant sociocultural influences. These findings complement RICH-LEGACY quantitative findings that documented high CVD risk factors in this population^16^ by illustrating the sociocultural influences on South Asian child and parent health behaviors.

Most participants were knowledgeable about the role of lifestyle factors in CVD risk. Yet it appeared that the knowledge about CVD risk did not necessarily translate into behavior change as evidenced by the number of children who self-assessed their diets as unhealthy. This apparent dissonance in knowledge and action when it comes to healthy eating has also been observed in Hispanic child and parent dyads^17^ and in white Canadian children.^18^ Social and cultural factors including peer influences in children, marketing of unhealthy foods to kids, and family dynamics that tend to influence cooking methods in South Asian households appear to be responsible for the observed dissonance. Consequently, given the array of influences on South Asian child and behaviors, it appears that interventions aimed at improving diets in children need to be multidimensional—considering the school environment, peer, and social media factors that shape children’s dietary habits.^19^

Perhaps, noteworthy are the subthematic differences observed between child and parent behavioral influences. Other than parental influences on food consumed at home, there appeared to be marked differences on the range of behavioral influences documented for children and parents. The impact of culture, especially as it relates to diet, was less salient for children. Children’s dietary and physical activity were influenced by their social environment (peers, school, and social media), whereas for parents, cultural factors and health reasons were the most important influences. These could possibly be as a result of acculturation differences where children of immigrant parents become immersed into the host culture more quickly than their parents.^20^ It is also possible that the observed differences might be generational, without links to culture, perhaps owing to changing social norms. Similarly, in a multiethnic qualitative study, Tiedje et al found that generational differences between adolescents and adults were more pronounced than observed ethnic differences.^21^ Consequently, these gaps in “deep structures” as described by Resnicow et al^13^ for children and parents suggest that potential interventions aimed at promoting lifestyle changes in South Asian populations must be individualized for children and parents.

Parental physical activity choices appeared to be more influenced by ease of participation and enjoyment of the activity rather than cultural relevancy, based on our data. This finding appeared to be consistent with other studies examining physical motivators in older individuals of other populations.^22^ Understanding the influence of these “surface structures” would be useful when designing interventions aimed at improving uptake in this population. We nevertheless found this result surprising as previous studies have shown that South Asians report cultural barriers to taking part in physical activity such as the inability to wear the religious dagger while swimming and were more open to culturally relevant activities such as yoga or Bhangra dance.^8,23^ It is possible that the same barriers reported for physical activity such as lack of time and injuries might also impede the adoption of culturally based physical activity like Bhangra dance that can be vigorous in nature. Evidence from this study suggests that physical activity seems individualized for the adult South Asian population; and thus, an understanding of individual context will be necessary when trying to design interventions to improve physical activity in the South Asian population.

### Implications for Practice

Overall, our findings provide insights into a range of behavioral influences on South Asian children and their parents. These findings provide noteworthy information that may be worth considering when designing interventions for this population. For example, strategies aimed at encouraging or improving healthy behaviors in children could take into consideration the range of influences such as the school environment, peer influences, and social media influences that positively or negatively influence dietary and physical activity habits. Given the ubiquitous use of social media in children, its use as a possible intervention tool in children might offer a cost-effective and innovative approach to improving dietary and physical activity–related behavior in children. However, care must be taken to avoid some of the pitfalls associated with social media use including its potential as an advertisement tool. Similarly, for South Asian parents, an understanding of motivators alongside barriers to dietary and physical activity–related behavior could be useful in the development of effective population-level health promotion or intervention plans aimed at promoting healthy behavior.

### Limitations

While the children included in the study were Canadian born, the parents in this study were a mix of Canadian-born and immigrant parents. To better contextualize the results presented, information around country of birth and time since immigration would have been useful. The absence of this information is a known limitation of this study. Additionally, while the sample size was sufficient enough to attain data saturation, the small sample size might limit the ability to obtain information from individuals with different sociodemographic characteristics thereby limiting transferability of findings.

## Conclusions

Despite the fact that studies have documented higher prevalence of CVD risk factors in South Asian children, qualitative research has been lacking on their attitudes and perspectives. We report findings from a qualitative study of South Asian children and their parents, documenting a wide array of factors that influence these attitudes and perspectives—and the related behaviors. We found that both children and parents demonstrated adequate knowledge about the role of diet and physical activity in CVD development. However, that knowledge did not always translate into healthy behaviors. For children, school, peers, and social media were observed to be the biggest influences on dietary and physical activity behaviors. For parents, culture and health-related factors were observed to be the biggest influences on dietary and physical activity behaviors.

Consequently, intervention strategies in the South Asian children and adult population must ensure that the range of social and cultural factors that influence behaviors—such as family, peer, and societal influences—are taken into account, if they are to yield meaningful and sustained outcomes.

## Supplemental Material

Supplementary_File – Supplemental material for Exploring Knowledge and Perspectives of South Asian Children and Their Parents Regarding Healthy Cardiovascular Behaviors: A Qualitative Analysis Click here for additional data file. Supplemental material, Supplementary_File for Exploring Knowledge and Perspectives of South Asian Children and Their Parents Regarding Healthy Cardiovascular Behaviors: A Qualitative Analysis by Adeleke Fowokan, Kaitey Vincent, Zubin Punthakee, Charlotte Waddell, Miriam Rosin, Navjot Sran and Scott A. Lear in Global Pediatric Health
