# Supplementary material for: Exploring Knowledge and Perspectives of South Asian Children and Their Parents Regarding Healthy Cardiovascular Behaviors: A Qualitative Analysis
Source: Glob Pediatr Health. 2020 Jul 1;7:2333794X20924505. doi: 10.1177/2333794X20924505 (PMC7331759; doi:10.1177/2333794X20924505)
Supplement: Supplementary_File – Supplemental material for Exploring Knowledge and Perspectives of South Asian Children and Their Parents Regarding Healthy Cardiovascular Behaviors: A Qualitative Analysis [file Supplementary_File.pdf]

Appendix 1

Interview Guide

Introductory question: In your opinion, what do you think people (both children and adults) can do to reduce their risk of heart disease?

South Asian's have been shown to be at increased risk for heart disease risk, and this risk has been shown to be present in children. What are some of the reasons you consider responsible for this?

Physical activity- Adults

- Physical activity plays an important role in reducing cardiovascular disease risk. As a result, we will like to seek your opinion on the following:
- What do you consider as exercise or physical activity? Please provide examples if you can.
- What kinds of physical activity/exercise would you be most likely to take part in? Why?
- What kinds of exercise are you least likely to participate in? Why?
- What do you consider the biggest barrier to being physically active?
- Are there any religious/cultural factors that tend to influence your decision to, or not to participate in physical activity?
- For those who engage in regular physical activity, what factors do you consider as motivators to participating in regular physical activity.
- Do you encourage your children to be physically active?

Children

- What does it mean to be healthy? What are some of the things you need to do to stay healthy?

- Do you think physical activity is important to the health of children?
- When are you the most physically active? School, home or with friends?
- Do you enjoy exercising or being physically active?
- Is there anything/ anyone that stops you from being physically active (TV, computer games, friends)

Nutrition- Adults

- Diet plays an important role to heart disease risk. As a result, we will like to seek your opinion on the following questions:
- Do you consider diet/nutrition as an important factor to attaining good cardiovascular health?
- What do you think healthy eating means?
- In your opinion, what should constitute a healthy diet? Does your diet meet these requirements?
- What is your general perception of the traditional South Asian diet?
- What are some of the qualities of the South Asian diet you think needs to be improved on to achieve good heart health?
- Do you read food labels before purchasing them from the grocery store?
- What are some of the barriers you and your family face to eating healthy?
- What are some of the things that motivate you to eating healthy?
- In your opinion, what are some of the things that can be done to increase awareness of healthy behaviours?
- Asides from healthy eating and exercise, what other thing do you think can help improve heart health or reduce your risk of heart disease?

Children

- What do you know about what makes a healthy diet?
- Do you think eating healthy now helps you when you get older?
- Are there any types of foods you are not allowed to eat?
- Where else do you eat apart from your home? And what kind of foods do you eat there?
- Are there any foods you think are not good for you? Give me examples.
- Would you say that your diet is healthy? What are some of the things that make you want to eat healthy?
- If your diet is not healthy, what stops you from eating healthy?
- Are you likely to eat healthier or exercise more if your favorite celebrity were doing same on social media? If yes, why?
